# The Role of Nerve Growth Factor (NGF) and Glial Cell Line-Derived Neurotrophic Factor (GDNF) in Tic Disorders

**DOI:** 10.12669/pjms.344.15555

**Published:** 2018

**Authors:** Ali Karayagmurlu, Hakan Ogutlu, Ibrahim Selcuk Esin, Onur Burak Dursun, Ahmet Kiziltunc

**Affiliations:** 1 Dr. Ali Karayagmurlu, MD. Consultant, Department of Child and Adolescent Psychiatry, Gaziantep Maternity and Child Health Hospital Gaziantep, Turkey; 2 Dr. Hakan Ogutlu, MD. Consultant, Department of Child and Adolescent Psychiatry, Erzincan University Faculty of Medicine, Erzincan, Turkey; 3 Ibrahim Selcuk Esin, Assistant Professor, Department of Child and Adolescent Psychiatry, Ataturk University Faculty of Medicine, Erzurum, Turkey; 4 Onur Burak Dursun, Associate Professor, Department of Child and Adolescent Psychiatry, Ataturk University Faculty of Medicine, Erzurum, Turkey; 5 Prof. Ahmet Kiziltunc, Department of Medical Biochemistry, Ataturk University Faculty of Medicine, Erzurum, Turkey

**Keywords:** Tic Disorder, Glial Cell Line-Derived Neurotrophic Factor (GDNF), Nerve Growth Factor (NGF), Neurotrophic factor

## Abstract

**Objectives:**

Tic disorders are associated with neurodevelopmental origin, changes in dopaminergic neurons, and the formation of immunoreactivity, it is thought that neurotrophic factors may be crucial in the emergence of tic disorders. In this study, we targeted to explore role of neurotrophic factors in tic disorders. The aim of this study was to investigate serum Glial Cell Line-Derived Neurotrophic Factor (GDNF) and Nerve Growth Factor (NGF) levels in patients with tic disorder and healthy controls.

**Methods:**

Thirty-four children, constituted the case group, were diagnosed with tic disorder. The control group included 34 healthy children. Development and Well-Being Assessment (DAWBA) (structured interview) and Yale Global Tic Severity Rating Scale (YGTSRS) was applied to the patients. NGF and GDNF levels were measured with ELISA kit.

**Results:**

In case group, serum NGF and GDNF levels were found to be significantly higher in females than males (p = 0.042, p = 0.031). It was determined that serum NGF and GDNF levels were correlated with each other (r = 0.803, p <0.001) and there were no correlations between other parameters. There was no significant difference in NGF and GDNF in patients with tic disorder, compared to healthy controls.

**Conclusions:**

The absence of this relationship does not exclude the hypothesis that neurotrophic factors may play a role in the etiopathogenesis of tic disorders.

## INTRODUCTION

Tics are sudden, rapid, non-rhythmic and repetitive movements or voices. Evidence has been established in recent years that hypersensitivity of postsynaptic dopamine receptors or excessive dopamine release takes place in the etiology of the disorder in individuals diagnosed with Tourette’s Syndrome (TS).^1^ The effectiveness of antidopaminergic agents in the treatment of tic disorder also supports this hypothesis.^2^ However, the course of tic disorders cannot be fully explained by this hypothesis.

Neurobiological factors are also implicated in the etiopathogenesis, depending on that the tic disorders are neurodevelopmental disorders. Neurodevelopmental disorders may occur due to problems with neurodevelopmental steps such as synaptic and neuronal growth myelinization, neuronal differentiation, and synaptic pruning. Neurotrophic factors play a major role in neurodevelopmental steps.^3^

In addition, immunoreactivity formation can occur through neurotrophic factors, and psychiatric disorders may occur. Neurotrophic factors act as reciprocal mediators between the neurons and the immune system cells to provide modulation of the immune system.^4^ There is evidence that immunoreactivity plays a role in the etiopathogenesis of autism spectrum disorder, chronic schizophrenia, and major depressive disorder.^5^-^7^ Because tic disorders are associated with neurodevelopmental origin, changes in dopaminergic neurons, and the formation of immunoreactivity, it is thought that neurotrophic factors may be crucial in the emergence of tic disorders.

Neurotrophic factors include Nerve Growth Factor (NGF), Glial Cell Line-Derived Neurotrophic Factor (GDNF), Brain-Derived Neurotrophic Factor (BDNF), neurotrophin-3 (NT-3), etc. NGF is a neurotrophic factor that has critical tasks in the development of the central nervous system. GDNF is another important neuropeptide for the development of dopaminergic and noradrenergic systems.^8^

In this study, we aimed to investigate the role of neurotrophic factors in the etiopathogenesis of Tic disorder. The purpose of this study was to compare serum GDNF and NGF levels, demographic characteristics and clinical parameters between in patients with tic disorder and healthy controls.

## METHODS

This was a cross-sectional case-control study. Ethical permission was obtained from the Ethical Committee of Atatürk University Medical School. Written informed consent was obtained from the parents accepted to participate in the study. The sample of the study consists of sixty-eight children in age range of 6-11 years. Thirty-four children, constituted the case group, were diagnosed with tic disorders at the first time at the outpatient clinic of Ataturk University Medical Faculty Child and Adolescent Psychiatry, and Gaziantep Maternity and Children Diseases Hospital, Child, and Adolescent Psychiatry. The control group included thirty-four healthy children without psychiatric and medical disease, applied to the outpatient clinic of Atatürk University Medical Faculty Pediatrics. There was no participant using drugs in both groups. All participants were assessed to have a normal level of cognitive development.

### Clinical Assessments

The sociodemographic data form containing information about the child and his or her parent was asked to be filled in for both groups participating in the study. A structured clinical interview, Development and Well-being Assessment (DAWBA) was conducted the parents of participants for the detection of psychiatric comorbidities. In addition, Yale Global Tic Severity Rating Scale (YGTSRS) was applied to the patients to determine the distribution and severity of tic disorder.

DAWBA is a structured diagnostic package that allows evaluation of common psychiatric disorders in children and adolescents based on DSM V. The validity-reliability study of DAWBA Turkish adaptation was reported by Dursun et al. and the results obtained from Turkish DAWBA are valid.^9^

YGTSRS: Motor and vocal tics are recorded in the last week with child and parent notification. Tics are graded on number, frequency, intensity, complexity, and interference.^10^ Intravenous blood sample was taken from participants to evaluate the neurotrophic factor levels. NGF and GDNF levels were measured with a Sun Red Biological commercial Human NGF and GDNF ELISA kit via a BioTek Power Wave XS micro plate reader.

### Statistical Analysis

Statistical analyzes were performed using Statistical Package for Social Sciences (SPSS) Statistics 23.0 program. For comparison of serum NGF and GDNF levels and non-normally distributed variates, Mann-Whitney U test; for comparison of normally distributed variates among the groups, Student t-test was used. Spearman correlation analysis was performed to determine the relationship between age, NGF and GDNF level, and tic disorder severity. Statistical significance level was accepted as p <0.05.

## RESULTS

### Demographic Data and Clinical Variables

Sociodemographic and clinical characteristics of the participants are shown in Table-I. There were no significant differences between these two groups with respect to age (p=0.149), gender (p=0.086), and maternal and paternal education levels (p=0.442, p=0.808, respectively).

**Table-I:** Sociodemographic and Clinical Features of Participants.

		n	%
Maternal Education Level	Highly Educated	23	33.8
	Lowly Educated	45	66.2
Paternal Education Level	Highly Educated	33	48.5
	Lowly Educated	35	51.5
Type of Tic	TS	12	35.3
	Motor	22	64.7
Comorbidity	Present	22	32.4
	Absent	46	67.6

### Serum NGF and GDNF Levels

Serum NGF and GDNF levels were not significantly correlated with gender (p = 0.236, p = 0.097), age (p = 0.756, p = 0.936), groups (p = 0.121, p = 0,783), maternal and parental education level (p = 0.055, p = 0.364; p = 0.153 and p = 0.927 respectively), type of tic (p = 0.958, p = 0.763) and comorbidity (p = 0.409, p = 0.829). In Table-II, demographic and clinical features were compared with the case group. In this comparison, serum NGF and GDNF levels were found to be significantly higher in females than males (p = 0.042, p = 0.031).

**Table-II:** Relationships between NGF and GDNF and Sociodemographic Data in Case Group.

		NGF				GDNF			
		Median	Minimum	Maximum	p	Median	Minimum	Maximum	p
Gender	Male	1051.25	533.75	3641.25	0.042^a^	15.31	6.94	75.36	0.031^a^
	Female	1488.75	653.75	3266.25		18.19	13.53	70.89	
Type of Tic	TS	1142.50	691.25	3266.25	0.958^a^	16.70	12.15	70.89	0.763^a^
	Motor	1210.00	533.75	3641.25		16.09	6.94	75.36	
Maternal Education Level	Highly Educated	1096.25	533.75	2801.25	0.209^a^	16.56	6.94	47.51	0.615^a^
	Lowly Educated	1337.50	653.75	3641.25		16.23	11.54	75.36	
Paternal Education Level	Highly Educated	1051.25	533.75	2731.25	0.106^a^	16.09	6.94	75.36	0.708^a^
	Lowly Educated	1321.25	653.75	3641.25		16.64	11.54	70.89	
Comorbidity	Present	1256.25	533.75	3641.25	0.763^a^	16.83	6.94	63.98	0.763^a^
	Absent	1072.50	716.25	3266.25		15.09	12.15	75.36	

a Mann Whitney U Test *TS: Tourette Syndrome, Motor: Chronic Motor Tic Disorder az

### Association between Serum NGF and GDNF Levels and Clinical Variables

The correlation between serum NGF and GDNF levels and age and tic severity scale were examined, it was determined that serum NGF and GDNF levels were correlated with each other (r = 0.803, p <0.001) and there were no correlations between other parameters (p> 0.05). Correlations are given in Table-III.

**Table-III:** Relationships between NGF and GDNF and Numeric Data.

	NGF		GDNF	
	r	p	r	p
Age	-0.038	0.756	0.010	0.936
Number of Tics	0.082	0.644	-0.091	0.608
Motor Tics Severity Score	-0.204	0.247	-0.248	0.157
Vocal Tics Severity Score	0.041	0.818	-0.135	0.447
Total Tic Severity Score	-0.044	0.803	-0.185	0.295
Total YGTSRS Score	0.028	0.875	-0.145	0.415
GDNF	0.803	<0.001	1.000	.
NGF	1.000	.	0.803	<0.001

*Spearman Correlation Analysis.

Tourette syndrome patients had significantly higher levels of total YGTSRS, total tic severity and vocal tic severity scores compared to patients with motor tics (p = 0.001, p=0,002, p = 0.015 respectively).

## DISCUSSION

Our study was perhaps the first study to investigate serum NGF and GDNF levels in tic disorder. There were no significant differences between case group and control group in NGF and GDNF levels.

There is a limited literature on the relationship between tic disorders and neurotrophic factors. In a family study of patients with TS, the genes on centromeric region of chromosome 5 were investigated and the relationship was not determined between GDNF and TS. In this study, although six strongest candidate genes were investigated in order to form neurodevelopmental basis of TS, all genes were found unrelated.^11^ Our study results did not show a significant relationship among GDNF and spectrum of tic disorders, including TS, indicates that similar results were obtained with this study.

Previously, there was not any neurotrophic factor study of tic disorder. The fact that NGF and GDNF levels were not correlated with tic disorders in our study does not exclude the role of neurotrophic factors in the etiopathogenesis of tic disorder. The main reason why this relationship cannot be detected in our study, the samples might not be taken from brain tissue, the main place of the disease.

Serum NGF and GDNF levels were higher in females with tic disorder than males in our study. As mentioned above, the level of serum NGF may be increased after the acute stress-induced reaction. Anxiety symptoms in females are more common than males; it can be attributed to the higher acute and chronic stress experienced by women.^12^ In our study, the presence of higher levels of NGF in female patients may be an indicative of the increased stress experienced by women. In a study investigating the relationship between anxiety symptoms and NGF gene, NGF C/C (Cytosine/Cytosine) genotype was found in women with high anxiety and men with low anxiety.^13^ It has been reported that this difference in females is related to reproductive hormones, estrogen has an effect on sympathetic system dependent on NGF and progesterone shows an anxiolytic effect, which is the contrasting effect of estrogen.^14^ In two studies with depression and OCD, it was shown that the GDNF level was not affected by gender. Unlike these results, GDNF levels were higher in women with tic disorder in our study.

We found a positive relationship between NGF and GDNF levels. Neurotrophic factors have vital functions in central nervous system tasks, for instance the functional maintenance of neurotransmitters and ion channels, axonal growth and dendritic pruning. It has been shown that neurotrophic factors include GDNF, NGF and BNDF work together in different pathways and in neurotransmitters such as dopamine and serotonin.^15^-^17^ This relationship obtained in our study can be explained by the fact that both parameters are neurotrophic factors, contribute to similar functions and work together.

There was no correlation between YGTSRS tic scale scores and NGF and GDNF levels in our study. While there is no study of NGF and GDNF in tic disorders, in a study investigated catecholaminergic genes there was a significant correlation between the BDNF gene expression and the severity of tic.^18^ In another study, there was no relationship between BDNF polymorphisms and tic severity.^19^ Similar to this study, no correlation was found between NGF and GDNF and tic severity, in our study.

### Limitations of the study

These include a low number of the participants and the samples were collected from peripheral blood rather than the brain tissue, which forms the pathology of the disease and that the parameters based on plasma levels instead of related genes. It is thought that the genes to be investigated in postmortem brain tissue samples may provide more reliable data.

In conclusion, there was no significant difference in serum NGF and GDNF levels in patients with tic disorder, compared to healthy controls. The absence of this relationship does not exclude the hypothesis that neurotrophic factors may play a role in the etiopathogenesis of tic disorders. Serum NGF and GDNF levels were higher in females with tic disorder than males. In addition, NGF and GDNF levels were correlated with each other. There is a need for studies to investigate the relationship between neurotrophic factors and tic disorders by increasing the number of samples and studying a number of genetic and biochemical parameters.
